# Symptomatic hypogammaglobulinemia in infancy and childhood – clinical outcome and in vitro immune responses

**DOI:** 10.1186/1471-2296-5-23

**Published:** 2004-10-21

**Authors:** Mona Iancovici Kidon, Zeev T Handzel, Rivka Schwartz, Irit Altboum, Michael Stein, Israel Zan-Bar

**Affiliations:** 1 Department of Pediatrics, Kaplan Medical Center, Rehovot, Israel; 2 Allergy and Clinical Immunology Unit, Kaplan Medical Center, Rehovot, Israel; 3 Department Human Microbiology, Sackler Faculty of Medicine, Tel Aviv University, Tel Aviv, Israel

**Keywords:** Humoral immunodeficiency, Transient hypogammaglobulinemia, Mitogens

## Abstract

**Background:**

Symptomatic hypogammaglobulinemia in infancy and childhood (SHIC), may be an early manifestation of a primary immunodeficiency or a maturational delay in the normal production of immunoglobulins (Ig). We aimed to evaluate the natural course of SHIC and correlate in vitro lymphoproliferative and secretory responses with recovery of immunoglobulin values and clinical resolution.

**Methods:**

Children, older than 1 year of age, referred to our specialist clinic because of recurrent infections and serum immunoglobulin (Ig) levels 2 SD below the mean for age, were followed for a period of 8 years. Patient with any known familial, clinical or laboratory evidence of cellular immunodeficiency or other immunodeficiency syndromes were excluded from this cohort. Evaluation at 6- to 12-months intervals continued up to 1 year after resolution of symptoms. In a subgroup of patients, in vitro lymphocyte proliferation and Ig secretion in response to mitogens was performed.

**Results:**

32 children, 24 (75%) males, 8 (25%) females, mean age 3.4 years fulfilled the inclusion criteria. Clinical presentation: ENT infections 69%, respiratory 81%, diarrhea 12.5%. During follow-up, 17 (53%) normalized serum Ig levels and were diagnosed as transient hypogammaglobulinemia of infancy (THGI). THGI patients did not differ clinically or demographically from non-transient patients, both having a benign clinical outcome. In vitro Ig secretory responses, were lower in hypogammaglobulinemic, compared to normal children and did not normalize concomitantly with serum Ig's in THGI patients.

**Conclusions:**

The majority of children with SHIC in the first decade of life have THGI. Resolution of symptoms as well as normalization of Ig values may be delayed, but overall the clinical outcome is good and the clinical course benign.

## Background

Pediatric patients with "recurrent infections" within our area are referred to the pediatric immunology clinic in the Kaplan Medical Center. Few fulfill the clinical criteria of the immune deficiency "red flags", Table 1, and only in a small minority, quantitative or qualitative defects in immunological function are documented. As expected, most such defects, involve the humoral immune system, the most common of the primary immune deficiencies [1,2]. Classically, the clinical presentation, includes a neonatal "grace" period, during which the baby is protected from infection by the presence of passively acquired maternal antibodies. As the level of these antibodies decline, the babies present at the end of the first year of life or the beginning of the second with recurrent respiratory, ENT and GI infections. The pathogens involved are mostly the "usual" bacteria, *Streptococcus pneumonia*, *Haemophilus influenza *and *Staphilococcus aureus*, but the infections may be of unusual severity, persistence, or frequency.

**Table 1:** Clinical "Red Flags" for Immunodeficiency

**1**	Eight or more new ear infections within 1 year
**2**	Two or more serious sinus infections in 1 year
**3**	Two or more months on antibiotics with no effect
**4**	Two or more pneumonias within one year
**5**	Failure of an infant to gain weight or grow well
**6**	Recurrent, deep skin or organ abscesses
**7**	Persistent thrush in the mouth or elsewhere on the skin, after age 1
**8**	Need for intravenous antibiotics to clear infection
**9**	One or more deep-seated infections such as sepsis, meningitis or cellulitis
**10**	A family history of primary immune deficiency or early infant death from infection, recurrent infection, malignancy, or autoimmune disease

Adapted from: Primary Immunodeficiency Diseases: A molecular and genetic approach New York, Oxford University Press, 1999.

In the last 10 years, tremendous advances in the fields of molecular medicine and genetics, have made possible the definitive diagnosis of most combined immunodeficiency patients, agammaglobulinemia patients and clinical syndromes associated immunodeficiency patients, on the basis of a recognized genetic aberration leading to a protein product dysfunction [3,4]. Nevertheless, the diagnosis of some of the most common forms of primary immune deficiency, IgA deficiency [5], common variable immunodeficiency [6] and transient hypogammaglobulinemia of infancy (THGI), are still based on clinical criteria and the exclusion of other specific diagnoses [7,8]. THGI is thought to be caused by a poorly understood maturation delay in the normal production of Ig, extending the physiologic hypogammaglobulinemia of the new born beyond the first year of life [1,9]. Currently, there are no diagnostic tests that differentiate, on initial presentation of a young child with recurrent infections and low Ig levels, those that will spontaneously correct on follow-up from those where a primary and permanent immune deficiency will develop, except for B cell numbers below 2%, which point towards X-linked agammaglobulinemia (XLA)[10].

In this study we aimed to evaluate the natural course of disease in symptomatic hypogammaglobulinemia of infancy and correlate in vitro lymphoproliferative and secretory responses to mitogens in this population with recovery of immunoglobulin values and clinical resolution.

## Methods

### Patients

Children more than 1 year of age, with recurrent infections, defined as more than three episodes of acute otitis media and/or more than one episode of acute sinusitis and/or more than one episode of pneumonia or the presence of a severe deep seated infection (meningitis, septicemia, etc.) within the last 6 months, or fulfillment of one of the "red flags" of immunodeficiency, see Table 1, and hypogammaglobulinemia, defined as serum Ig values 2 SD below the age defined norms on two or more measurements [11], have been prospectively recruited from a cohort of children referred to our clinic because of recurrent or severe infections. Patients were seen at presentation and reevaluated periodically at 6 to 12 month intervals, up to 1 year after resolution of symptoms.

All procedures were performed according to accepted ethical standards of the Institutional Review Board of Kaplan Medical Center. The parents of all the children were informed accordingly and gave their permission for participating in the study and blood sampling.

### Ig assessment

Total serum Ig levels were measured by nephelometry (Beckman Immunochemistry Systems, IgM, IgG and IGA test, Beckman Instruments, Galway, Ireland) and serum IgG subclasses have been assayed with an immunodiffusion commercial kit (Human IgG Subclasses single dilution BINARID, Birgminham, UK). Specific antibody production was not evaluated.

### In vitro cell proliferation and Ig secretion

Peripheral blood mononuclear cells (PBMC) were isolated from heparinized venous blood of patients and age-matched donors (children hospitalized or attending the outpatient clinic for unrelated conditions) on ficoll isopaque gradients (Sigma, St. Louis, MO, USA). Patient and normal donor cells were cultured in microtiter plates with culture medium: RPMI medium supplemented with 10% FCS (Bio-Lab, Jerusalem, Israel), 10 mM Hepes buffer, 100 U/ml penicilin 100 μg/ml streptomycin, 2 mM L-glutamine, and 100 μg/ml kanamycine (Sigma Israel), and were grown at 37°C with 7.5% CO2 in air. 5 × 10^5 ^cells were stimulated for four days with 0.01% w/v SAC (Calbiochem, La Jolla, CA, USA), 2.5 μg/ml PWM, 20 μg/ml E. coli: O55:B5 LPS or with 20 μg/ml PHA (Sigma, St. Louis, MO, USA). The cells were then pulsed with 1 μCi/well of [^3^H]-Thymidine (Nuclear Research Center, Negev, Israel) and incorporated radioactivity was measured by a β scintillation counter. Proliferation was expressed as stimulation index (SI).

In parallel, cell culture supernatant aliquots were harvested and Ig isotype concentrations were measured in the culture supernatants, by a solid-phase immunoassay, in Nunc- Immunoplate Maxisorp 96 wells (Nunc, Denmark). The plates were coated with goat anti-human IgM, IgG or IgA antibodies (Jackson Immunoresearch Laboratories, West Grove PA, USA). Biotinilated goat anti-human IgM, IgG or IgA (Jackson, West Grove, PA, USA) and streptavidin-alkaline phosphatase (Amersham, Buckinghamshire, England) were used for measurement of IgM, IgG and IgA respectively. Resulting yellow dye intensity was read by an ELISA reader (Microplate Auto-Reader Bio-Tek Instruments, VT, USA). Dye units were converted to immunoglobulin concentrations by extrapolation from standard curves determined by using purified myeloma proteins of known concentration in every assay.

### Statistical analysis

We used non-parametric tests to compare the means (Kruskal Wallis test for k independent samples) and a standard analysis of variance to compare between groups. Logistic regression analysis was used for comparison of distribution of dichotomous values between the groups. Analysis was performed using SPSS for windows ver 9.0.

## Results

32 patients were included in the study, 24 males (75%) and 8 females (25%) with a mean age at diagnosis of 3.4 years (range 1.2 – 7.0). Clinical presentations included severe and recurrent Ear-Nose-Throat (ENT) infections – 22 patients (69%), pneumonia, bronchopneumonia or severe, recurrent upper respiratory infections – 26 patients (81%), diarrhea – 4 (12.5%) and atopy related complaints – 20 patients (63%). A positive family history of recurrent, unusual or severe infections was obtained in 7 patients (22%). Demographical and clinical data of patient cohort is summarized in Table 2.

**Table 2:** Clinical and Demographic Data of Patient Cohort

		**THGI**	**Non Transient**	**p**
Demographics	No of Patients	17 (53%)	15 (47%)	> 0.05
	Males	15 (88%)	9 (60%)	> 0.05
	Females	2 [30]	6 [31]	> 0.05
	Average age at Diagnosis	3.6 years	3.1 years	> 0.05
Clinical Data	ENT	10 (59%)	12 (80%)	> 0.05
	Respiratory	15 (88%)	11 (73%)	> 0.05
	GI	2 [32]	2 [33]	> 0.05
	Atopy	11 (65%)	9 (60%)	> 0.05
	Family History	4 (24%)	3 (20%)	> 0.05
Diagnosis	IgAD	9 (53%)	7 (48%)	> 0.05
	IgGD	12 (71%)	10 (68%)	> 0.05
	IgMD	2 [34]	3 (20%)	> 0.05
Treatment	Antibiotics	4 (24%)	8 (53%)	> 0.05
	IVIg	2 [35]	1 [36]	> 0.05
Outcome	No Infections	14 (83%)	13 (87%)	> 0.05
Follow-up (years)	Age at last follow-up	7.1	5.5	> 0.05
	Length of follow-up	3.5	2.5	> 0.05

THGI – Transient Hypogammglobulinemia of Childhood

Non Transient – Hypogammaglobulinemic patients who did not correct during follow-up

IgAD – IgA 2SD bellow age specific norms

IgGD – One or more IgG isotype 2SD bellow age specific norms

IgMD – IgM 2SD bellow age specific norms

Out of the initial 32 patients, 17 (53%) spontaneously corrected their Ig abnormalities. This group included 15 boys (88%) and 2 girls[12], mostly in the second or third year of life, average age at diagnosis being 3.6 years (range 1.3–9). The two groups, those with essentially transient hypogammaglobulinemia – THGI and those who did not correct their Ig values during the follow up period did not differ significantly at diagnosis, see Table 2.

All defects were partial, no patient in this group, showing a complete absence of a given Ig isotype. The clinical course was benign, only 9.4% (3/32) patients requiring IVIg (2 in the THGI group, 1 in the non corrected group). 38% (12/32) of patients received prolonged antibiotic prophylaxis (4/17 in the THGI and 8/15 in the non corrected group, p = 0.09) and resolution of clinical symptoms occurred in 84% of patients (14/17 in the THGI and 13/15 in the non corrected group). All calculated p values, non significant for comparison between the 2 groups.

Comparative analysis of serum Ig isotype levels at diagnosis showed no significant difference, between the transient and non-transient group.

**Figure 1:**
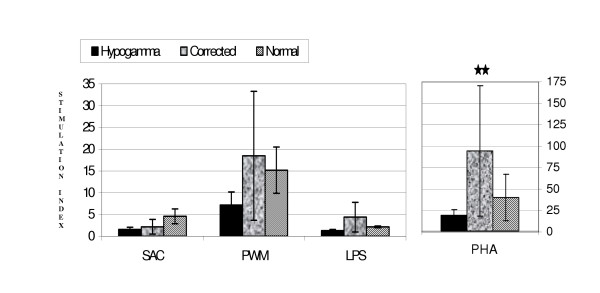
**Lymphocyte proliferation after in-vitro mitogenic stimuli in Pediatric Hypogammaglobulinemia. **SAC – Staph. Aureus Cowan. PWM – Pokeweed Mitogen PHA – Phytohemmaglutinin LPS – Lipopolysacharide Values are given as Stimulation Index (SI) mean ± 95% CI, ** p < 0.005

In vitro lymphocyte proliferation and Ig secretion were measured in 9 patients (5 patients with THGI and 4 with non-corrected IGD) on one or more occasions. Lymphocytes proliferative responses to SAC, PWM and LPS showed no differences between the groups and no significant differences from childhood norms. The proliferative response to PHA was significantly increased (p < 0.005) after the correction of Ig abnormalities, overshooting normal controls (Fig 1).

Quantification of the in-vitro immunoglobulin secretion in response to the various mitogens showed significant isotype and mitogen dependent variation. The IgM secretory response to PWM and LPS, was low in hypogammaglobulinemic patients. After normalization of serum Ig values, the IgM response to LPS stimulation increased, see table 3. However, the improved response to LPS was still lower than age matched controls (p < 0.05).

**Table 3:** Immunoglobulin Secretion from B-cells after in-vitro mitogenic stimuli in Pediatric Hypogammaglobulinemia

	**SAC**			**PWM**			**LPS**		
	**IgM**	**IgG**	**IgA**	**IgM**	**IgG**	**IgA**	**IgM**	**IgG**	**IgA**
**Hypogamma**	1.6	1.2 *	1.1 **	1.1 *	1.4 *	1.3 *	1.3 *	2.0 *	1.1 *
	(0.6–2.6)	(0.9–1.5)	(1.0–1.2)	(0.9–1.3)	(0.9–1.9)	(1.0–1.6)	(1.1–1.5)	(1.1–2.9)	(1.0–1.2)
**Corrected**	2.8	1.0 *	1.3 *	1.0 *	1.1 **	1.3 *	2.2 *	1.5 *	1.2 *
	(1.7–3.9)	(0.9–1.1)	(1.0–1.6)	(0.9–1.1)	(1.0–1.2)	(1.1–1.5)	(1.4–3.0)	(1.2–1.8)	(1.0–1.4)
**Normal**	3.0	2.5	2.3	2.6	3.0	2.2	4.4	5.0	3.4
	(2.5–3.5)	(1.9–3.1)	(1.8–2.8)	(2.2–3.0)	(2.4–3.6)	(1.7–2.7)	(3.9–4.9)	(3.5–6.5)	(3.0–3.8)

SAC – Staph. Aureus Cowan. PWM – Pokeweed Mitogen

LPS – Lipopolysacharide

Values are given as Secretion Index Mean and (95% confidence interval)

** p < 0.005 compared to normal controls

* p < 0.05 compared to normal controls

For both IgG and IgA, the response in normal children was significantly better than in either group of patients. The IgA secretion index to all 3 stimulatory mitogens was minimal in hypogammaglobulinemic patients, even after serum Ig correction and differed significantly from age matched controls (p < 0.005 for SAC, p < 0.05 for PWM, p < 0.05 for LPS), table 3. The IgG secretion index in hypogammaglobulinemic patients was slightly better to LPS than to other mitogens (non significant) and differed significantly from age matched controls (p < 0.05 for SAC, p < 0.005 for PWM, p < 0.05 for LPS). No difference was observed on either test between THGI and non corrected patients on initial presentation.

## Discussion

Young patients with recurrent infections represent a sizeable portion of the daily practice of all primary care pediatricians and family physicians, with parents clamoring for a solution with the accumulation of lost daycare or school days. The physician is faced with the dilemma when parental assurance will suffice, rather than initiation of a costly immunological investigation. Often, the presenting clinical signs and symptoms are insufficient for an educated diagnosis, as well as Ig levels in infants below the age of one year. The present investigation was initiated in order to try to contribute additional understanding how to differentiate between cases of primary immune deficiency and those who are not.

We prospectively studied the outcome of SHIC in 32 patients during a period of 8 years, mean follow up of 3.2 years. During this time more than half corrected their Ig abnormalities. The mean follow up of the non-corrected IGD group is slightly shorter (though not statistically significant) than that of the THGI group (2.5 years and 3.5 years respectively) which leads us to speculate that some patients in the "non-transient" group may eventually correct. This is consistent with previously published reports from Dalal et al. who, after a follow up of 10 years, found that 70% of patients had complete resolution of their Ig abnormalities [13].

Interestingly, the majority of our patients were males (75%), and a higher proportion of males corrected their serum Ig (15/24 males and 2/8 females). This finding of male preponderance is not uniquely ours. In the reports of Dalal et al. [14], 24/35 (69%) and Walker et al. [15] 29/39 (74%) of patients included in the study were male. We found no readily available explanation for this phenomenon.

It is impossible to estimate the prevalence of THGI in Israel, from our data, since our patients are part of a biased referral clinic population. Still, our impression is that this diagnosis is perhaps the most common of the Ig deficiencies in childhood, an impression supported by a number of publications from different groups [16,17].

In the paragraph on THGI in the most recent Scientific Group Report on the subject [1], the age cutoff of recovery of normal Ig synthesis capability "may be delayed for as long as 36 months". In contrast, in our series of patients, normalization to age adjusted Ig levels, has occurred in some cases at 8 or 9 years of age. These results are consistent with Dalal et al [18], who also describe resolution of THGI over the first decade of life. A more recently published series [19], 7/40 [20] of patients with THGI, still had low levels of antibodies at age 5 years. More over, the benign course of the group of patients who did not correct their Ig values during the course of our follow up, may indicate that a significant portion of these patients may actually still belong to the THGI group and will correct on subsequent follow up. These combined observations may induce a change in the classical definition and diagnostic criteria for THGI [21].

The majority of patients (84%) resolved their tendency for recurrent infections irrespective of their Ig values. Only a minority of patients required any medical intervention, 38% received antibiotic prophylaxis and only 9% intravenous Ig replacement therapy. We observed no difference in the clinical presentation or follow up of the transient group as compared to the non corrected IGD patients and resolution of serum Ig abnormality did not cause a complete clinical remission in all patients. This may be due to a residual inability to mount an adequate antibody response to specific antigen challenge, data that has not been evaluated in our series. The impaired IgG and IgA in vitro secretory responses, seen in hypogammaglobulinemic patients, even after serum Ig normalization, may be an expression of such an inability, which warrants further investigation.

Atopy was a prominent associated complaint in 21/37 (57%) of our patients. Especially so in comparison with the prevalence of asthma in this age group in Israel – 7% [22,23] and the estimated prevalence of other atopy associated diseases, about 20%. This finding was inconsistently reported by other investigators [24,25].

Though no clinical evidence of T-cell functional impairment was observed in our patients (no opportunistic, fungal or chronic viral infections), previous reports [26-28] suggest that the apparent B cell defect may be secondary to a T cell dysfunction such as T cell cytokine disregulation. We have shown that cellular and humoral responses to mitogenic stimuli tend to be lower in SHIC patients as compared to normal children but do not differentiate THGI from patients with more persistent hypogammaglobulinemia. Lymphocyte proliferation in response to PHA is significantly increased after Ig correction in THGI, overshooting normal controls. The molecular basis of this observation is unclear. In vitro IgM secretion is less impaired than other isotypes and in THGI a definite improvement of IgM secretion with SAC and LPS stimulation occurs concomitantly with correction of serum Ig. The IgG and IgA secretion in response to mitogenic stimuli is severely impaired and does not normalize concomitantly with serum Ig, indicating a possible impairment in the isotype switching mechanism. This observation is supported by the previous long term follow-up reported by Dalal [29] where specific IgG antibody responses to polysaccharide antigen were reduced even after the resolution of the serum Ig deficiency in a large subgroup of patients with apparently resolved THGI.

## Conclusions

THGI is a relatively common cause of symptomatic hypogammaglubulinemia in infancy in our area. Most children will spontaneously correct their Ig abnormalities during the first decade of life. Though tests of cellular or humoral stimulation index, are not as yet capable of differentiating the transient from the non-transient patients upon their presentation, significant isotype and mitogen specific variability is evident. The relative preservation of the in vitro IgM secretory response and the lack of IgA/IgG response in patients with hypogammaglobulinemia, argues for a delay in isotype switching as the molecular basis underlying the clinical entity of transient hypogammaglobulinemia of infancy.

## Competing interests

The author(s) declare that they have no competing interests.

## Abbreviations

SHIC – Symptomatic Hypogammaglobulinemia in Infancy and Childhood

THGI – Transient Hypogammaglobulinemia of Childhood

Ig – Immunoglobulin

IGD – Imunoglobulin deficiency

SAC – Staphylococcus aureus cowan I

PWM – Pokeweed mitogen

LPS – Lipopolysaccharide

PHA – Phytohemagglutinin

IVIg – Intravenous Immunoglobulin

ENT – Ear, nose & throat

## Authors' contribution

MIK – carried out the patient care and follow-up, was responsible for the database organization, data analysis and for manuscript coordination and writing

ZT – is one of the research initiators, carried out the patient care and follow-up and contributed to the manuscript writing

IA – carried out the in vitro cell proliferation and Ig secretion studies

RS – carried out the in vitro cell proliferation and Ig secretion studies

MS – carried out the patient care and follow-up, was responsible for the database initiation

IZ – is one of the research initiators, carried out of the laboratory evaluation and contributed to the manuscript writing

All authors read and approved the final manuscript

## Pre-publication history

The pre-publication history for this paper can be accessed here:


